# Elicitation of trustworthiness requirements for highly dexterous teleoperation systems with signal latency

**DOI:** 10.3389/fnbot.2023.1187264

**Published:** 2023-08-23

**Authors:** Joe Louca, John Vrublevskis, Kerstin Eder, Antonia Tzemanaki

**Affiliations:** ^1^Bristol Robotics Laboratory, University of Bristol, Bristol, United Kingdom; ^2^Advanced Concepts Team, Thales Alenia Space, Bristol, United Kingdom; ^3^Trustworthy Systems Laboratory, University of Bristol, Bristol, United Kingdom

**Keywords:** telemanipulation, trustworthiness, human-robot interaction, user requirements, space robotics, delayed teleoperation, trust

## Abstract

**Introduction:**

Teleoperated robotic manipulators allow us to bring human dexterity and cognition to hard-to-reach places on Earth and in space. In long-distance teleoperation, however, the limits of the speed of light results in an unavoidable and perceivable signal delay. The resultant disconnect between command, action, and feedback means that systems often behave unexpectedly, reducing operators' trust in their systems. If we are to widely adopt telemanipulation technology in high-latency applications, we must identify and specify what would make these systems trustworthy.

**Methods:**

In this requirements elicitation study, we present the results of 13 interviews with expert operators of remote machinery from four different application areas—nuclear reactor maintenance, robot-assisted surgery, underwater exploration, and ordnance disposal—exploring which features, techniques, or experiences lead them to trust their systems.

**Results:**

We found that across all applications, except for surgery, the top-priority requirement for developing trust is that operators must have a comprehensive engineering understanding of the systems' capabilities and limitations. The remaining requirements can be summarized into three areas: improving situational awareness, facilitating operator training, and familiarity, and easing the operator's cognitive load.

**Discussion:**

While the inclusion of technical features to assist the operators was welcomed, these were given lower priority than non-technical, user-centric approaches. The signal delays in the participants' systems ranged from none perceived to 1 min, and included examples of successful dexterous telemanipulation for maintenance tasks with a 2 s delay. As this is comparable to Earth-to-orbit and Earth-to-Moon delays, the requirements discussed could be transferable to telemanipulation tasks in space.

## 1. Introduction

Trust is essential for effective collaboration and optimal performance in human-robot teams (Chen et al., 2018). This is particularly the case in space for human-robot teams that involve teleoperated systems over long distances, which result in lengthy signal delays (Lester and Thronson, 2011). These delays create a disconnect between the operator sending a command, the remote system taking action, and, subsequently, the operator receiving feedback. Therefore, as latency increases, the operator must delegate greater responsibility to the robot to carry out actions successfully, without supervision. In order to overcome this cognitive barrier, the operator must *trust* that the robot will complete the task without errors, and the robot must demonstrate to the operator that it is *trustworthy*.

There are several definitions of *trust* and *trustworthiness* reported in the literature with respect to human-robot teams (Abeywickrama et al., 2023). In this work, we have used the following definitions:

- *Trust*—willingness to rely on the system, based on confidence that it will behave as expected (Chopra and Wallace, 2003; Schaefer, 2013).
- *Trustworthiness*—assurance that a system will behave as expected (Avizienis et al., 2001).

Trust evolves as a function over time (Hoff and Bashir, 2015; Chen et al., 2018). It is hard to gain, can easily be lost, and is subsequently far more difficult to regain (Lee and See, 2004). In contrast, trustworthiness is an inherent quality of a system which must be demonstrated to the user (Abeywickrama et al., 2023).

While a system might be technically safe and effective, it will not be put to use unless the users perceive it to be trustworthy (Abeywickrama et al., 2023). Despite trust being an evolving, subjective variable, we can identify the conditions under which a user will place their trust in a system, i.e., what is required for the system to be worthy of the user's trust. Provided we have accurately captured what is required to make a system trustworthy, we can demonstrate that systems meet these requirements to ensure they are verifiably trustworthy (Abeywickrama et al., 2023).

Previous work has identified non-functional requirements which ensure reliability (Gupta, 2022) or safety (Dede et al., 2021) in systems involving a human-robot team. While these aspects are related to a system's trustworthiness, they do not consider the user's perspective. Agrawal et al. (2020) considered trust when specifying the requirements for human interaction and intervention points in a system involving multiple robots, but this focused on the relationship with semi-autonomous aerial vehicles rather than robot manipulators with low levels of autonomy. To our knowledge, there have been no previous studies discussing the requirements for demonstrating trustworthiness in teleoperated robot manipulators, also known as *telemanipulation*.

For the foreseeable future, a subset of tasks in space will require direct human intervention due to their complexity and uncertainty (NASA, 2010; Smisek, 2017). As a result, operators may need to adjust their commands based on their observations, and, hence, direct teleoperation would be preferred, as opposed to using automated control (Nair et al., 2020). Directly teleoperated systems with a human-in-the-loop are more closely supervised by the operator, and, consequently, establishing trust in these systems requires a different approach from those with greater degrees of autonomy (Nahavandi, 2017). With this in mind, the scope of this paper is focused on systems involving telemanipulation.

For decades, telemanipulation has been used in a range of applications with high safety or financial risks, for example, robot-assisted surgery (RAS; Gharagozloo et al., 2021), nuclear reactor maintenance (Moore, 1984), underwater exploration (Anderson, 1960; Wang and Cui, 2021), and ordnance disposal (Hallett and Weedn, 2016). The fact that, across these application areas, operators willingly transfer accountability to their robots to carry out duties on their behalf, demonstrates their trust in the system. Despite the clear benefits and use cases (Grandl, 2007; Howe and Colombano, 2010; NASA, 2010; Xue et al., 2021), this trust in telemanipulation systems has not yet been widely extended to the space environment. There are still very few examples of direct telemanipulation with delays over long distances in space (Henshaw et al., 2022), even though the technology has been mature for some time (Canadian Space Agency - Government of Canada, 2018).

The literature suggests various technical approaches which could improve performance of delayed telemanipulation systems (Beik-Mohammadi et al., 2020; Panzirsch et al., 2020; Pryor et al., 2020) but there has been limited other research investigating whether any of these features demonstrate trustworthiness Rogers et al. (2017). As trust is a human quality and, therefore, its perception depends on the human user of a system, user-centric and non-technical approaches are necessary for its development. In this paper, we explore how trust is inspired in existing telemanipulator systems, through a series of interviews with expert operators. We identify what leads users to trust their systems in well-established example applications, in order to develop a set of user requirements for a trustworthy telemanipulation system. Furthermore, we compare the challenges posed by delay in these applications against those for the space environment to determine which features or practices could be transferred across domains.

## 2. Methods

We conducted 13 interviews with participants with telemanipulation experience in four target application areas: four nuclear reactor maintenance, three underwater exploration and maintenance, three robotic explosive ordnance disposal, three RAS surgeons. These application areas were selected as well-established examples of where direct teleoperation has been implemented. They cover a range of task types, which incorporate a range of similar challenges to those encountered in the space industry. For example, operating with high precision during surgery, or as a free body when underwater. Crucially, each application has a considerable safety or risk component, with the potential for negative consequences as a result of errors, and therefore requires trust from the operator. Participants were recruited through a combination of social media advertising, direct emailing, and mailing lists to members of specific organizations (e.g., The Institute of Explosives Engineers). The criteria for selection were that they must regularly operate remote machinery which includes a manipulator arm. Their mean age was 44.9 years, with 1:12 ratio of female to male participants, which was the maximum ratio achieved during the 18 month span of the project. The participants represented commercial, academic, and government organizations based in the UK and USA. We assigned ID codes to identify the responses of different individuals in this report: N1-4 for Nuclear operators, U1-3 for underwater operators, O1-3 for ordnance disposal operators, and S1-3 for the RAS surgeons. These IDs are used in Table 1 to describe the spread of experience levels across our sample and outline the applications and systems used for each participant. The descriptions of applications and systems are intentionally broad in order to protect the respondents' identity, as the community of robot operators is small. The study was approved by the Faculty of Engineering Research Ethics Committee of the University of Bristol (ID: 2021-0204-262).

**Table 1:** Academic backgrounds and system information for each participant.

**ID**	**Highest academic qualification achieved**	**Experience**	**System information**	**Application**
N1	National vocational qualification level 3	Mid-career	Dual arm manipulators with force feedback and articulated boom	Nuclear decommissioning
N2	National vocational qualification level 3	Senior	Dual arm manipulators with force feedback and articulated boom	Nuclear decommissioning
N3	BEng automotive engineering	Junior	Dual arm manipulators with force feedback	Nuclear decommissioning and reactor maintenance
N4	Higher national diploma mechanical engineering	Mid-career	Dual arm manipulators with force feedback	Nuclear decommissioning/Reactor maintenance
U1	BSc marine science	Senior	Remotely operated vehicles with dual arm manipulators	Ocean science/Engineering
U2	Ph.D.	Junior	Remotely operated vehicles with dual arm manipulators	Ocean scientific research
U3	M.Sc	Senior	Remotely operated vehicles with single and dual arm manipulators	Marine science and maintenance
O1	CEng, CMarEng	Senior	Various remotely operated systems with single or dual arm manipulators	Bomb and mine clearance, Firefighting, nuclear and hazardous material handling, underwater systems
O2	[Declined to answer]	Senior	Various mobile systems with single or dual arm manipulators	Explosive ordnance disposal robots
O3	Level 7 Diploma	Mid-career	Various mobile systems with single arm manipulators	Explosive ordnance disposal robots
S1	Bachelor of medicine, bachelor of surgery	Senior	Da Vinci surgical system	Robot-assisted colorectal surgery
S2	Bachelor of medicine	Senior	Da Vinci surgical system Renishaw neuromate ROSA robot	Robot-assisted neurosurgery
S3	Bachelor of medicine	Senior	Globus excelcius robotic navigation platform	Robot-assisted spinal surgery

Semi-structured interviews were conducted over video-calls to address three research questions (RQ):

- RQ1: Tolerable Delay—What delay is currently tolerated in telemanipulation systems, and what features or techniques help to overcome this problem?
- RQ2: Time Dependent Actions—On what timescale do operators need to respond to events?
- RQ3: Trustworthiness Requirements—What features, techniques or experiences build trust in telemanipulation systems?

The interviewer followed a protocol consisting of six major questions, with a mixture of open and closed sub-questions, to form the basis of the discussions:

1. *Can you give a brief overview of your telemanipulation system and application area? What do you consider to be the main advantages and disadvantages of your system as it is now?*
2. *When operating your system, do you feel confident that the machine will perform as intended? Why is this? Is this based on features, proofs, or experience?*
3. *Have you noticed any delay when operating your system? How do you deal with it?*
4. *Can you give any examples of decisions or actions you have to make which are time sensitive? On what timescale do these happen?*
5. *Can you envisage any situations where you would rather the machine takes the lead? What would give you confidence in relinquishing control? What would stop you?*

*Question 6* presented the participants with the following examples of features which could be included in telemanipulation systems to aid the operator:

- Fixed or variable camera viewpoints.
- Virtual/augmented/mixed reality.
- Different types of haptic feedback—force/cutaneous/ vibrotactile.
- Direct camera streams or representative virtual views of the scene.
- High resolution video.
- High frame rate video.
- “Ghost Mode” display of predicted actions.
- Virtual fixtures outlining “no-go zones,” communicated through visual, audio, or haptic feedback.
- Waypoint marker displays to communicate task progress.

Participants were asked to discuss any of these, or any others which they had encountered, that stood out as particularly useful, or not useful, and why. This part of the interview was left as open as possible in order to avoid prescribing solutions to the operators, as is best practice in requirements elicitation (Goguen and Linde, 1993). Across all questions, the interviewer also asked follow-up questions based on the responses to explore topics in greater detail, whilst letting the interviewee lead the discussion.

Answers to simpler questions (i.e., closed questions or shorter open questions) were collated and directly compared. *Questions 3* and *4* were used to inform *RQ1* and *RQ2*.

*RQ3* was addressed by performing open coding thematic analysis over the entirety of the discussions, with particular focus on *Questions 1, 2, 5*, and *6* (Merriam and Tisdell, 2015). We assigned words, phrases, or sentences in order to reduce the raw data into named, meaningful segments, to enable comparisons between the various phrasings and terminologies used in different conversations. For example, “we build physical mock-ups to practice on” and “training is carried out using cadavers” from separate interviews would both assigned the segment “Operators train on physical replicas.” A priority score, based upon MoSCoW prioritization (Hatton, 2007), was then assigned to each of these segments based upon the context of the participants' responses. This placed each segment into one of four priority categories. In decreasing order of priority, these are: “Must have” (M) = 3 points, “Should have” (S) = 2 points, “Could have” (C) = 1 point, and “Will not have” (W) = −3 points.

**Figure 1:**
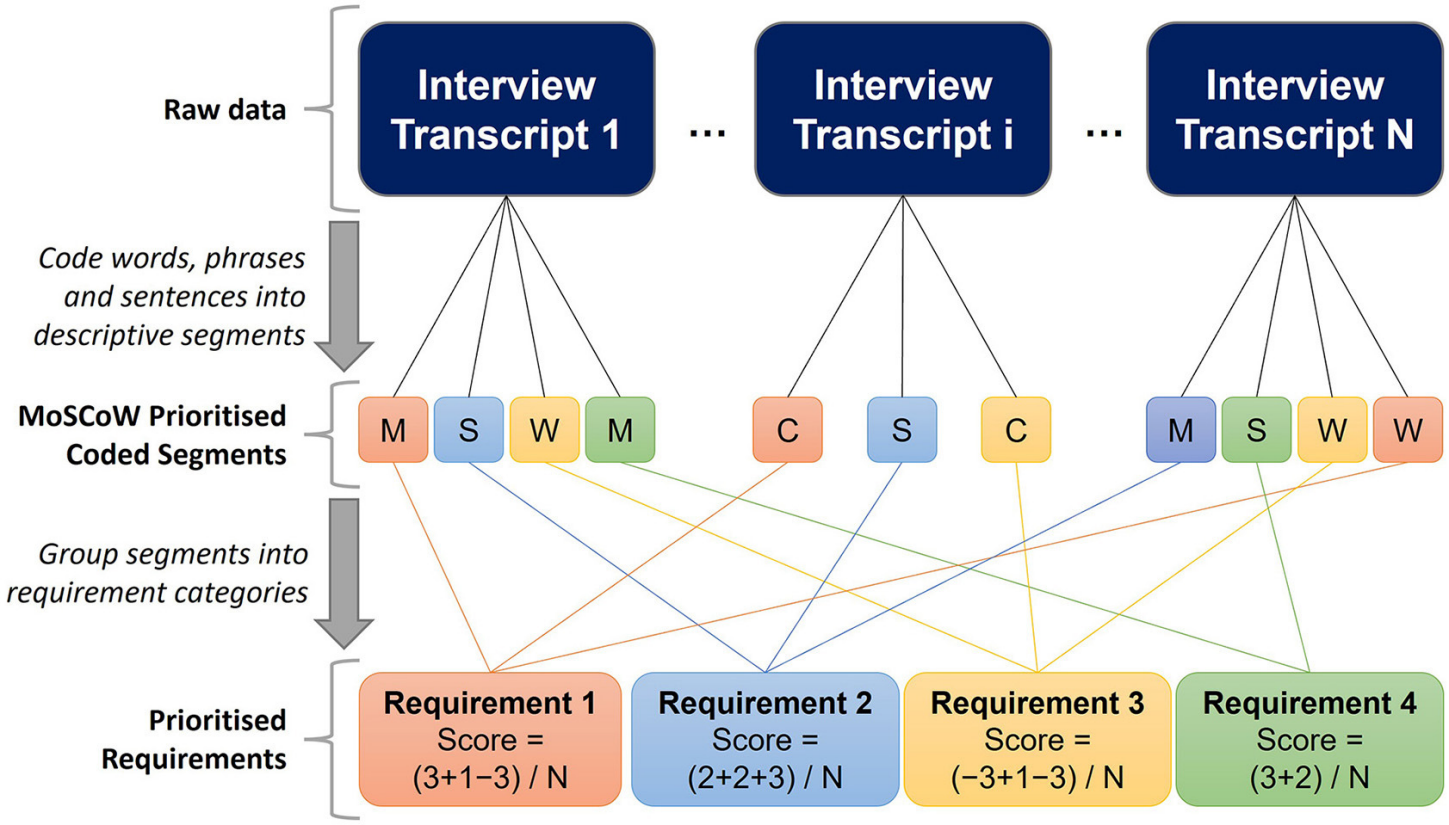
Illustrative example of the open coding thematic analysis procedure used to analyse interview data to address RQ3. M, “must have” (3 points), S, “should have” (2 points), C, “could have” (1 point), W, “will not have” (−3 points). Segments are grouped into requirement categories (different colors), which are subsequently scored as the sum of segment scores divided by the number of interviewees. Requirement scores > 2: Mandatory requirements. Scores ≥ 1.5: High-priority requirements. Scores ≥ 1: Medium-priority requirements. Scores ≤ 0: Low-priority requirements.

The collection of segments were then grouped into categories, i.e., a list of requirements for trustworthiness, via a combination of inductive and deductive thinking. From this, a total score was calculated for each category by summing the scores across all interviews and dividing by the number of participants in that category. Mandatory requirements were identified as those with a total score >2, i.e., those that were majority “must have.” High-priority requirements were identified as those with a total score ≥1.5. Medium priority requirements were identified as those with a total score >1. Components to avoid (negative requirements) were identified as those with a total score ≤ 0. This procedure is illustrated in Figure 1.

Responses were firstly analyzed “within-case,” considering only the responses from participants from the same application area (Ayres et al., 2003). The same analysis was then carried out “across-case” to identify the trends which applied more generally. The raw, anonymized interview data will be available in an online repository with the final version of the paper.

## 3. Results

In this section, we describe the results of the interviews structured in the context of the three research questions outlined above, and subsequently by application area. Direct quotes from the interviews have been provided in quotation marks, and are attributed to individuals by their ID in brackets. Where phrases are attributed with no quotation marks, the authors have paraphrased or summarized the participants' words for brevity and clarity.

### 3.1. RQ1—Tolerable delay

Table 2 summarizes the degrees of delay tolerated by each participant for their applications. These delays ranged from 0 to 2 s for direct teleoperation, with larger delays accepted when using higher-level control modalities.

**Table 2:** Degrees of delay tolerated in teleoperation systems by each participant.

**ID**	**Tolerated delay**	**Comments**	**Application**
N1	None perceived	Any delay is tuned out during development.	Nuclear decommissioning
N2	None perceived	−	Nuclear decommissioning
N3	None perceived	≤ 200 ms delay for the digital twin.	Nuclear decommissioning and reactor maintenance
N4	None perceived	−	Nuclear decommissioning/reactor maintenance
U1	None perceived	Has experienced larger delays when communicating with a telepresent command team. 7–15 s for low level commands 24 h delay for high level objectives.	Ocean science/engineering
U2	1–2 s (optical link) 1 min (acoustical link)	Optical link used for general piloting of the ROV. Manipulation is difficult but possible. Acoustical link limits control to safety checks and high level commands only.	Ocean scientific research
U3	None perceived (usually) 2 s (currently, due to a fault)	Operators adapt to the larger delay with experience. Maintenance tasks, such as replacing components and mating connectors, are possible with 2 s delay.	Marine science and maintenance
O1	≤ 200 ms	−	Bomb and mine clearance, nuclear and hazardous material handling
O2	None perceived	−	Explosive ordnance disposal robots
O3	None perceived (fiber optic) ≤ 1 s (wireless)	−	Explosive ordnance disposal robots
S1	None perceived	−	Robotic colorectal surgery
S2	None perceived	−	Neurosurgery
S3	None perceived	−	Spinal surgery

#### 3.1.1. Nuclear

There was no noticeable delay on the example systems from the nuclear domain, as any latency had been “designed out” during development (N2). This was common across all operators interviewed apart from one exception when discussing operation of N3's manipulator via a virtual model. The visualization of this model updates at 5 fps, resulting in delays up to 200 ms delay on the virtual representation. N3 reported that this mode of operation is never used without close supervision, with operators remaining ready to press the emergency stop switch if there is significant deviation from what is expected. Additionally, the virtual representation is only used for large movements of the manipulator in free space to approach the target, not to interact with objects.

Despite having little experience using delayed systems, the operators speculated on the challenges this would present—“dexterous tasks would be really difficult” (N1)—and the approach they would take to overcome these. They reported that they could adapt to delays by adjusting their movement styles to use either: move-and-wait command profiles (previously reported by Sheridan and Ferrell, 1963), slower movements, or more pre-programmed or automated movements. In response to moderate delays, they suggested they could “move in small increments” (N2), or “make smaller moves, and then wait until you get the feedback, and then make another move and wait for the feedback. So it would be much slower.. you couldn't operate the equipment in a fluid sense” (N4). Whereas with large delays, they would “move [the arm] to pre-programmed points one at a time.” (N2), or “use pre-programmed arm moves that would get you close [to the start position]” (N4). It is important to note that, just like controlling a manipulator via a virtual representation, they specified that automated or pre-programmed movements must be used exclusively for approaching targets and not for interacting with them. Interviewees were “happy in instances where the kit is in control, but not when it comes to actually doing the task” (N1), where they require full control of the system. N1 gave the example of “screwing in a nut,” where, “especially at the start, you need to find out if you've cross-threaded it... You need to be able to feel that it's going in correctly.” Participants were comfortable using pre-programmed movements for “large movements between tasks” in free-space and “anything repeatable” (N1), to get up to get to the point where you need to take control.” N3 described what would give them sufficient confidence to execute a pre-programmed movement. Moves are all “created by individuals such as myself, all reviewed secondarily by a reviewer, and they have been cleared and marked as safe. They have been released so you have some confidence that the move has already been checked.”

#### 3.1.2. Surgical

S1-3 all reported zero noticeable delay in their telemanipulation systems. However, this was not the case for the first example of long-distance telesurgery, between Strasbourg and New York (Marescaux et al., 2001). The mean total time delay in this system was 155 ms, whereas the limit of the acceptable time delay with regards to safety, reported by surgeons, was 330 ms. While they did not have first-hand experience of this, S2 referred to another example of long distance telesurgery that was successfully performed from London, Ontario to Halifax, Nova Scotia, Canada (2,848 km round-trip), with 370 ms delay (Nguan et al., 2008).

#### 3.1.3. Underwater

Participants from the underwater domain reported a range of communications delays with their systems. The biggest factor impacting delay in this application was the communication type. Real-time control enabled by the use of fiber optic cables was generally preferred by all three underwater operators: they operate their systems via tethering cables several km long instead of via an optical link from the shore which would result in a 1–2 s delay (U2). Piloting the robot through the latter method is possible, but manipulation tasks become difficult. Acoustical communications allow users to communicate with the remote system over long distances and when tethering is not practical (U2). Delays in this mode can be up to 1 min due to the need for signal scheduling to avoid send-and-receive crosstalk. In this mode, operation is limited to making safety checks and sending high level commands only.

Two particularly novel examples of delayed teleoperation were described by the underwater operators. U3 reported that, while usually they experience little to no delay in their telemanipulation tasks, they had recently been operating their manipulator with a variable 2 s delay due to a fault. They found that operators were able to adapt to this larger delay after some time operating the faulty system, and were capable of carrying out their usual maintenance tasks such as replacing components or mating connectors.

U1 had experience operating their system to follow commands via video link from telepresent subject matter experts on the shore, resulting in a 7–15 s variable delay. In this situation, the operator described themselves (paired with the robot) as the “remote system” which received instructions from the command team as the “local system.” Delayed commands from the shore pertained to high-level objectives, and the operator was piloting the craft as a teleoperated mobile robot. This delay did not impact telemanipulation tasks. As the delays often resulted in overshooting objectives, the operators adapted by reducing their moving speed to make response times more appropriate for the delay. They even trialed this in the extreme case of a 24 h delay in receiving commands from the shore. Operating effectively under this level of communication delay required clarity of instructions from the local command team, combined with a more detailed contextual understanding of the mission by the operators at the remote end—“it actually turned into a bit more of a human experiment than an experiment with the robots because it was, it became more about communication protocols and how people interpreted what the actual commands were” (U1).

#### 3.1.4. Ordnance disposal

As was the case in the underwater domain, the delay experienced by ordnance disposal operators was dependant on the communication mode. Using a fiber optic connection resulted in zero noticeable delay (O2, O3). Real-time operation made this the preferred mode of operation, even taking into account the practical drawbacks of using a tethered robot. In wireless mode, however, delays could be up to 1–2 s (O3).

### 3.2. RQ2—Time dependent actions

Operators were required to be more reactive in environments with greater uncertainty, and they described several examples of actions that must be taken which are time-dependent. The preferred method of dealing with these actions was prevention. This was achieved, firstly, by identifying areas of uncertainty in the task, and then, reducing these uncertainties where possible. Uncertainties could be identified and reduced by providing additional viewpoints to the operator, modeling target objects in advance to make their behavior more predictable, or reducing the complexity of the robot itself. Areas with remaining uncertainties in the task were addressed through operator training and experience.

#### 3.2.1. Nuclear

The highly controlled environments of the reactor maintenance examples discussed meant that the vast majority of tasks did not require the operator to be reactive. The entire reactor configuration was known and any objects were fixed down when not in use so that there would be no unexpected movements. The participants believed that over the lifetime of the system, any areas of uncertainty have been identified and modeled in order to progressively reduce the number of unknowns in the environment. For example, bending cables can produce unexpected movements, which is why this has been modeled extensively to make the complex behavior more predictable (N1). Furthermore, the majority of tasks were set up to avoid any time pressure to reduce the chance of mistakes (N1).

While unexpected behaviors are largely avoided by having a tightly controlled environment, in some situations operators still needed to be reactive to unexpected situations resulting from their own limitations. After a camera view has been rotated, participants reported losing their perspective of gravity. This is important as when re-engaging force feedback, if the operator does not brace in the correct direction against the pull of gravity, they can easily allow the remote manipulator to fall. In these situations operators would have <1 s to respond to their error before colliding with the scene (N3).

#### 3.2.2. Surgical

Surgical environments are, by nature, more variable, and uncertain. One time-dependant action that surgeons must take is responding to bleeding, which must occur on the seconds-scale to avoid further complications (S1 and S2). Magnified camera views is an important feature in responding quickly to bleeds, as it enables more detailed inspection of the environment—“Because it's magnified, you can see a better view where the bleeding is... I move straight to [the bleed site] and hold it with the robot, so the response is actually quite quick” (S1).

#### 3.2.3. Underwater

In sub-sea applications, pressing the emergency stop button during automated procedures would be an example of a time-sensitive action (U1). The required response time for this would be around 2–3 s. The task environment dictates the severity of the risks, and, therefore, determines how crucial it is for operators to respond in a timely manner. For example, in exploration tasks to “pick up some small rocks... I wouldn't say it's delicate. It's pretty robust, so we know that the arm can be pretty rough” (U2). Furthermore, “most of the problems that would occur would be the arm damaging the ROV (remotely operated underwater vehicle)... There's nobody down there, so we can be a little more generous with our [response times]” (U1). The absence of humans or infrastructure, as well as the robustness of the environment, mean that the biggest risk would be colliding with the telemanipulator itself.

In infrastructure servicing tasks, however, the financial cost of damaged components makes it more important for operators to respond promptly to approaching collisions (U3). The 2 s delay discussed in Section 3.1.3 meant that operators needed to react in a timely manner to unexpected manipulator movements, such as overshooting the target when connecting sensitive components. This is overcome through training and practice under the challenging conditions.

#### 3.2.4. Ordnance disposal

For the majority of the time in ordnance disposal tasks, objects in the scene are stationary and, hence, do not need reacting to (O2). However, timely adjustments by the operator are necessary in some situations to effectively interact with objects. For example, opening car doors to access devices is a common task in ordnance disposal (O3). Making the initial grasp on a stationary door is a static interaction with the object, however, as the door opens the interaction becomes dynamic and therefore grasps can slip, which require the operator to reposition the manipulator (O3). Operators must detect and respond to this in <1 s. If the operator is attempting this with the 1–2 s delay (Section 3.1.4), they might not “notice it until [they have] already slipped, when [they] could have prevented that” (O3). The participant mentioned two ways to overcome this problem. The first was experience-based—“Once you've got more experience using [the system] you know whether you've got a good grip of the door”—and the second was based on simplifying the system—“The fix for that was, literally, a crowbar sellotaped and zip-tied to the end of the block on the top of the weapon, and then you just use the crowbar and you hooked [the door] and pulled it out.” Combined with the additional time constraints imposed by both the battery capacity of the manipulator and the configuration of the ordnance device, ordnance disposal operators must work under significant time pressure. Operators overcome this by following standard operating procedures for given tasks, in which they have high confidence that the procedure will be effective. O2 summarized this by saying “A lot of this is going to come down to procedures rather than technology, and it's the process of getting from an unknown situation to a place where we're confident that we could do whatever job it is.” Interviewees believed that the additional time spent during training to make these procedures automatic reduces the time spent on decision making during operations.

### 3.3. RQ3—Trustworthiness requirements

**Figure 2:**
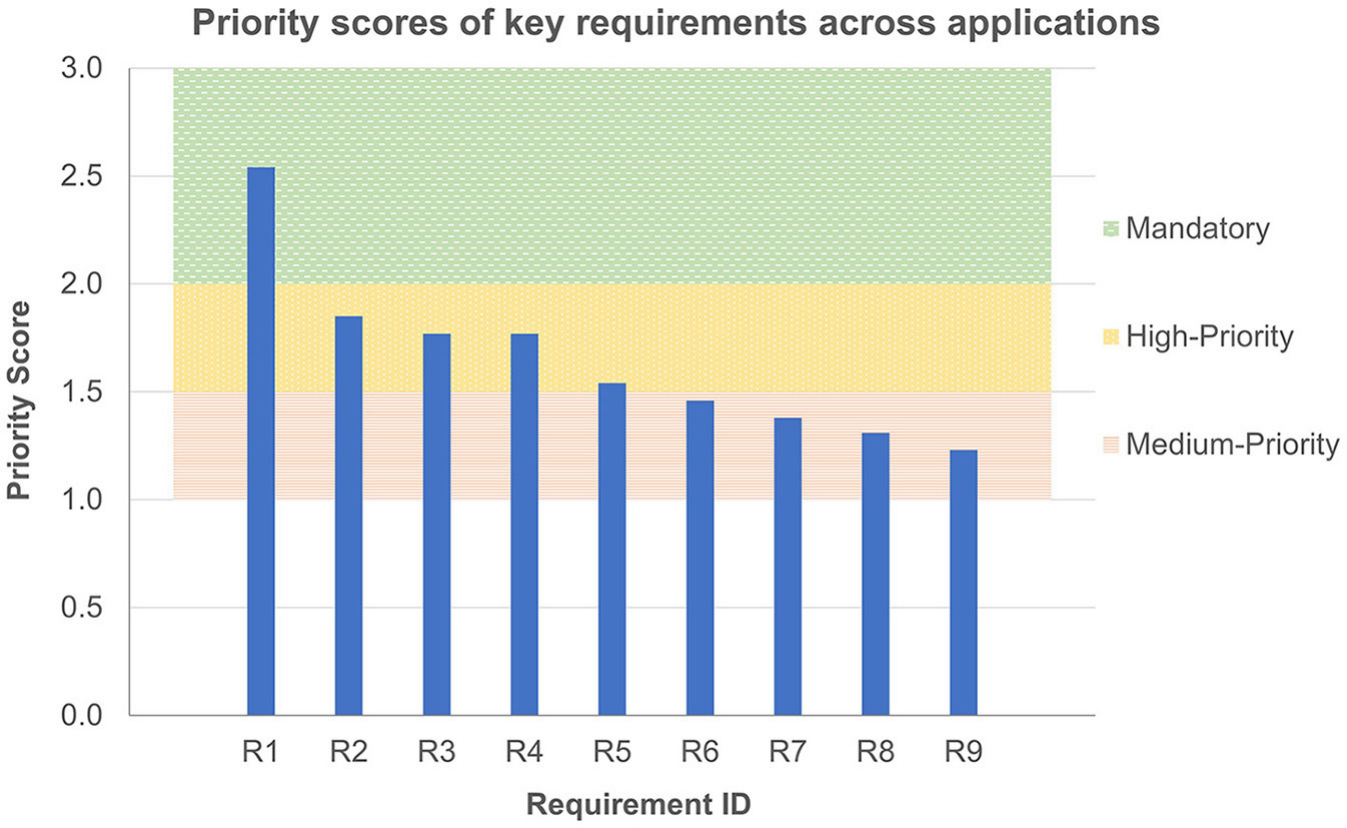
Priority scores of the mandatory, high-priority, and medium-priority requirements elicited from interviews across nuclear, surgical, underwater, and ordnance disposal applications. Full descriptions of requirements for each ID can be found in Table 7.

The open coding thematic analysis for the within-case perspectives generated sets of key requirements for trustworthiness, which are collated in Tables 3–6 for the nuclear, surgical, underwater, and ordnance disposal domains, respectively. The highest priority trustworthiness requirements for operators across all domains are collated in Table 7. Each row of these tables represents a requirement of the system discussed in the interviews that leads to greater operator trust. The whole system is considered, including the operator and any supporting elements, as well as the teleoperated device itself. Each requirement is ranked by a score calculated using the methodology of Section 2. The MoSCoW prioritization levels assigned during individual interviews is indicated in the right-hand columns. Figure 2 depicts the priority scores of the key requirements across all applications (Table 7), and their classification as mandatory, high-priority, or medium-priority requirements.

**Table 3:** Mandatory and high-priority requirements elicited from interviews with operators from nuclear reactor maintenance applications.

**Trustworthiness requirement**	**Score**	**N1**	**N2**	**N3**	**N4**
Operators have a comprehensive engineering understanding of the systems' and tools' capabilities and limitations.	2.25	m	m	m	−
Operators have experience operating the system with a low frequency of faults and uncontrolled or unrequested movements	2.25	m	−	m	m
System has “Hard” physical stops, which are irreversible and cannot be overridden	2.25	m	m	−	m
Operators are provided with camera views from fixed viewpoints for a consistent task overview perspective.	2.25	m	m	−	m
New components are tested virtually through simulations.	2.25	s	m	s	s
System includes a support team to spread out responsibilities beyond just the operator.	2.00	s	m	m	−
Operator display includes a “Ghost mode” to visualize manipulator movements ahead of execution.	1.75	s	s	m	−
Operator display includes a view of a virtual representation of the scene with as much detail as possible (<20 mm variance vs. real world), through which to carry out larger free-space movements or to plan actions offline.	1.50	−	s	s	s
Operators are provided with force feedback.	1.50	−	s	s	s
Force feedback magnitude can be adjusted for the individual operator and the task.	1.50	−	s	s	s

Right-hand columns indicate the assigned MoSCoW prioritization level: m, “must have,” s, “should have,” c, “could have.”

**Table 4:** Mandatory and high-priority requirements elicited from interviews with operators from surgical applications.

**Trustworthiness requirement**	**Score**	**S1**	**S2**	**S3**
Operators understand the systems capabilities, limitations, and idiosyncrasies.	3.00	m	m	m
Systems have accredited safety checks from a reputable authority to build and rebuild trust quickly.	3.00	m	m	m
Operators can measurably compare the robot's perceived state and its actual physical state.	2.00	−	m	m
System has an interrogable operating system that reports the live physical configuration, system health status, and decision reasonings.	1.67	−	m	s
Controllers and artificial constraints smooth out operator commands.	1.67	m	s	−
Clear, high-resolution images of the entire system in the scene.	1.67	m	s	−
Notify users of the cause and revert to known, safe states in response to errors.	1.67	s	m	−

Right-hand columns indicate the assigned MoSCoW prioritization level: m, “must have,” s, “should have,” c, “could have.”

**Table 5:** Mandatory and high-priority requirements elicited from interviews with operators from underwater exploration and maintenance applications.

**Trustworthiness requirement**	**Score**	**U1**	**U2**	**U3**
Clear communication with the support team to spread out responsibilities beyond just the operator.	3.00	m	m	m
Operators have a comprehensive engineering understanding of the systems', tools,' and support team's capabilities and limitations.	2.00	m	m	−
Systems are tested firstly in software, then in a physical mock up, then in the real world application, with increasing risk and realism.	2.00	−	m	m
Response protocols to known failure modes are planned, defined and trained for.	2.00	m	−	m
Operators have patience and vigilance when operating with delays.	2.00	−	m	m
Operators make slow movements when operating with delay.	1.67	s	−	m
Operators are provided with multiple camera views.	1.67	m	−	s
System fails to a safe state, using software as a first line defense, followed by mechanical backups.	1.67	m	s	−
Operators are provided with visual and audio feedback on system health status.	1.67	−	m	s
Operators have a large number of hours practice and experience with the particular system, focusing on operational time with a low frequency of faults or uncontrolled or unrequested movements, rather than on skills and techniques.	1.67	s	−	m
Simulation environments are used for training and testing.	1.67	−	s	m

Right-hand columns indicate the assigned MoSCoW prioritization level: m, “must have,” s, “should have,” c, “could have.”

**Table 6:** Mandatory and high-priority requirements elicited from interviews with operators from ordnance disposal applications.

**Trustworthiness requirement**	**Score**	**O1**	**O2**	**O3**
Operators have a comprehensive engineering understanding of the systems' and tools' capabilities and limitations.	3.00	m	m	m
Operators are provided with camera views from fixed viewpoints for a consistent task overview perspective.	2.00	−	m	m
Operators are provided with visual and audio feedback warnings on system health status.	1.67	m	−	s

Right-hand columns indicate the assigned MoSCoW prioritization level: m, “must have,”, s, “should have,” c, “could have.”

**Table 7:** Mandatory, high, and medium-priority requirements elicited from interviews across nuclear, surgical, underwater, and ordnance disposal applications.

**ID**	**Trustworthiness requirement**	**Score**	**Responses**
R1	Operators have a comprehensive engineering understanding of the systems', tools' and support team's capabilities and limitations.	2.54	N1-m, N2-m, N3-m, S1-m, S2-m, S3-m, O1-m, U2-m, O1-m, O2-m, O3-m
R2	Systems are tested firstly in software, then in a physical mock up, then in the real world application, with increasing risk and realism.	1.85	N2-m, U2-m, U3-m, O2-m, N1-s, N3-s, N4-s, S2-s, S3-s U2-s
R3	Operators are provided with multiple camera views from fixed and movable viewpoints, adequate to see all details of the scene.	1.77	N1-m, N2-m, N3-m, S1-m, S2-m, U1-m, O3-m, U3-s
R4	Health status monitoring systems send warnings to operators via visual and audio feedback, and provides documentation which explains the cause and effect of errors.	1.77	U2-m, O1-m, N4-m, S2-m, N1-s, N2-s, S3-s, U3-s, O3-s, N3-c
R5	Operators have a large number of hours practice and experience with the particular system, focusing on operational time with a low frequency of faults or uncontrolled or unrequested movements, rather than on skills and techniques.	1.54	N1-m, N3-m, N4-m, S2-m, O2-m, U3-m, U1-s
R6	Safety systems trigger in a fail-safe mode when outside the system's capabilities, using software as a first line defense, followed by mechanical backups.	1.46	N1-m, N2-m, N4-m, U1-m, O3-m, S1-s, U2-s
R7	Operators are provided with camera views from fixed viewpoints of the entire system for a consistent task overview perspective.	1.38	N1-m, N2-m, N4-m, S2-m, O2-m, O3-m
R8	Operators communicate clearly with a support team to distribute responsibilities and limit workloads.	1.31	N2-m, N3-m, U1-m, U2-m, U3-m, N1-s
R9	Operators are provided with high image resolution and frame rate camera views.	1.23	N2-m, S1-m, U2-m, O2-m, N1-s, N3-s

Right-hand column indicates the assigned MoSCoW prioritization level by each participant: m, “must have,” s, “should have,” c, “could have.”

System features which were negatively rated by at least one participant across all domains are collated in Table 8 and ranked inversely by their score, with the lowest priority (most unwanted) requirements at the top.

**Table 8:** Lowest priority requirements elicited from interviews with operators from across all applications.

**Trustworthiness requirement**	**Score**	**Responses**
System is controlled via a virtual representation.	−0.54	O3-s, N1-w, O1-w, O3-w
Operator uses a VR headset.	−0.46	N1-w, N4-w
System is capable of changing the end-effector tool automatically.	−0.08	N1-s, O3-w
System provides haptic feedback (in delayed systems).	0.00	N2-s, N3-s, S1-s, U2-s, O2-c, N4-w, U3-w, O3-w

Right-hand columns indicate the assigned MoSCoW prioritization level: m, “must have,” s, “should have,” c, “could have,” w, “will not have.”

## 4. Discussion

### 4.1. Delay and its effect on real-world operation

The level of acceptable delay varies widely across applications, although, unsurprisingly, lower delays are always preferable. The risks associated with the applications and the availability of other solutions had the most impact on this perspective. For instance, underwater operators were more accepting of delays in their systems because the consequences of errors are mild—U2 viewed their arm as “pretty robust” and U1 identified that “most of the problems that would occur would be the arm damaging the ROV... There's nobody down there,” which reduces the risks. Where there was an operational imperative, for example, teleoperating from the shore (1–2 s delay) or switching to long range acoustical communications (1 min; U2), operators would adapt to the more challenging conditions. This could be by switching to sending higher level commands, or, as in the examples reported by U2 and U3, direct telemanipulation was possible for exploration and maintenance tasks, but it required patience from the operator. Adaptability was also seen in ordnance disposal examples, where operators would accept a 1 s delay in cases where wireless control was essential. In contrast, the operators from nuclear and surgical domains unanimously stated that having near real-time control was an essential requirement, due to the high cost of failure. Some of these interviewees stated that they would refuse to use their system if it involved delays, as they would not be confident that it would behave as expected, i.e., they would not trust their system. This aligns with prior reports from the literature, particularly for bilateral haptic telemanipulation systems (Sheridan, 1993), as were used by N1-4.

The mixed reactions of interviewees in response to haptic feedback (Table 8) are also seen in the literature. Although haptic feedback improves transparency and the operators' situational awareness (Hoff and Bashir, 2015; Babarahmati et al., 2020), operators may be more tolerant of delays in systems without haptic feedback. For example, although Ivanova et al. (2021) demonstrated performance benefits by adding haptic feedback with delays ≤ 540 ms, the magnitude of this benefit reduced as latency increased. Interviewees from the underwater and ordnance disposal domain do not use haptic feedback, and were more accepting of small delays, <2–3 s, if it provided a practical advantage such as wireless operation.

While many of the systems discussed in the interviews were designed to reduce these delays to imperceivable levels, the signal propagation time in long-distance teleoperation cannot be entirely “designed out” (Lester and Thronson, 2011). Underwater teleoperation systems provide the closest terrestrial analog, with respect to delays, of teleoperation from Earth to orbit, or beyond. Our participants with underwater experience reported that it is possible to operate with a variable latency up to 2 s, comparable to Earth-Moon delays (2.6 s), for similar maintenance tasks to those in space, such as in-orbit servicing and maintenance (IOSM). Their system did not utilize any extra technical features to inspire confidence, but relied on the operator's understanding of the capabilities and limitations of their system after spending several hours on operating that specific system. We can, therefore, conclude that the human is the most adaptable node of a teleoperation system with today's technology, and systems should be developed to exploit this capability. Operator training that ensures complete familiarity with their systems should, therefore, be prioritized over the incorporation of new technical features, in order to develop trust. This agrees with reports from the literature on the effects of latency training and performance. Xu et al. (2015) found that training with 600 ms latency led to improved operator performance, and that it lasts at least one week, promising that operators can effectively adapt to higher latencies. The literature suggests a variety of technical approaches which could be utilized to mitigate the challenges of delayed teleoperation, with respect to performance (Babarahmati et al., 2019; Beik-Mohammadi et al., 2020). However, our results show that whilst these might be functionally capable, new technical features are not what experienced users of these systems would prioritize. Instead, the focus should be on user-centric solutions such as providing training which develops the operator's understanding and familiarity of their system. Technical solutions may offer assistance, but they must be introduced gradually to progressively demonstrate their effectiveness to the operator in situations with increasing risk and realism.

The analysis of the interviews showed that, across applications, operators needed to take action in response to events within 0–3 s. Providing that the round-trip latency of the system does not exceed the time required for any time-dependant action to be taken, we can assume that direct telemanipulation will be an acceptable control mode. No time-dependant actions were reported that would require response times greater than the expected delays for Earth-Orbit communication (100's ms; Lester and Thronson, 2011). Activity on the Lunar surface is likely to be more difficult as the communication delay (2.6 s) is comparable to some of the reported reaction times. Although a comprehensive review of potential IOSM and Lunar surface actions would be required to confirm this, our conclusions suggest that direct telemanipulation should be a viable option for future missions, particularly in orbit.

### 4.2. Requirements for developing trust

#### 4.2.1. Mandatory requirements

Our results in Section 3.3 identified one stand-out mandatory requirement to build human-robot trust in telemanipulator systems.

- Operators must have a comprehensive engineering understanding of the system's capabilities, limitations, and idiosyncrasies (R1).

Understanding of mechanical, electrical, electronics, and communications subsystems are all needed to build situational awareness, which lets the operator understand how the system will behave (U1). This understanding is what “differentiates between a user and a good user” (O1). This key finding aligns with and elaborates upon previous results from a survey of human-robot interaction, robotics and engineering experts who reported “situational understanding” as the most important factor in delayed teleoperation systems (Wojtusch et al., 2019). Here, we further identify the components required for the operator to achieve this situational understanding.

Although this view was not shared by the surgeons, they were still interested in understanding their system's functionality—“Each system comes with its own training and limitations, so you have just to know the system” (S1)—rather than understanding the mechanisms behind this. S2 clarified this point further, identifying that, where they do not have the engineering understanding of their system, this requirement is met vicariously by believing that the system providers have this expert knowledge instead. This “starts with reputation [of the system provider's organization],” and when adopting new systems, they “nearly always have the experts with the company down to show [surgeons] how to use it or troubleshoot it. Then if we run into difficulties, we largely accept it's going to do what it's meant to do... That's a very powerful thing.” S3 also shared this point of view, reporting that they often have an engineer, who is an expert in that system, supporting the surgeon to troubleshoot errors and explain the root causes. The difference between attitudes of the surgeons when compared with the other participants may be due to their background, which can influence how trust is developed (Nahavandi, 2017). In the three other categories, operators were generally trained engineers, which differs greatly to a surgeon's training. For space applications, as operators might have a more similar background to the nuclear, underwater and ordnance disposal experts, it is assumed that this requirement will also apply (Menchaca-Brandan et al., 2007).

It is expected that the human-robot relationship will evolve over time, with periods of increased or reduced trust in the system (Hoff and Bashir, 2015). As per the definition of trust in Section 1, uncertainty and perturbations from the environment will increase the likelihood of the system behaving unexpectedly, and will, subsequently, reduce trust. Similarly, witnessing errors or faults in the system will result in reduced confidence that it will behave as expected, also reducing trust. Being able to rebuild damaged trust and maintain it, therefore, is as important as building it in the first place. Having a holistic understanding of the system helps to explain unexpected events, which gives confidence that they can be avoided in the future. Trust in uncertain situations is based on the operator being able to “take steps from the information given to make some assumptions or at least an idea of what the vehicle is trying to do” (U2). To achieve this, operators need to be provided with sufficient information from which to predict the system's behavior.

#### 4.2.2. High-priority requirements

In addition to the mandatory requirement of the previous section, we identified four additional high-priority requirements from the results in Section 3.3, which can be grouped into: “improving situational awareness” and “facilitating operator training and familiarity.”

##### 4.2.2.1. Improving situational awareness

- Systems must provide a combination of fixed viewpoints, which provide an overview from a constant perspective, and variable cameras, which can move and/or zoom to provide detail on specific aspects of the scene (R3).
- System health monitoring systems must clearly communicate the occurrence, cause and effect of faults (R4).

Although many sensory feedback modalities were discussed, such as audio, forces, and vibrations which are often explored in the literature (Giri et al., 2021), the most important of these was having multiple camera viewpoints. The key aspect of this requirement is that the visual feedback system must be adequate to see any detail in the scene that may impact the task. These camera views could be supported by other sensory feedback types such as force feedback, but these were non-essential and application-specific. For example, operators from the nuclear domain relied heavily on force feedback, but this was not required by any other participants. Similarly, although using artificial intelligence to identify key features and augment camera views of the scene is an active area of research (Shi et al., 2023; Tian et al., 2023), this was seen as a low priority feature and was only mentioned by two operators (S1, N1). These responses were surprising, given the positive reports of these technical features in the literature, and highlight the need to provide operators with features that they actually require.

One form of feedback that was desired across applications was a health monitoring system which, not only identifies the system status, but also communicates the reasoning behind this. For example, N2 stated, “We have a lot of a diagnostic tools on the robots and the manipulators and they say, ‘Oh well, it's done that because of this.'... so I gained my trust,” and N3 said, “You'll get a pop up window on the HMI (human-machine interface) which will tell you are going to violate the safe margin.” Similarly, O3 described the need for warnings so that operators could prevent errors before they occur—“[The system] needs a good warning... If it had gone yellow, and then given you 2 or 3 s, then you have to let go of the system before going on it again to go a bit further, that would have been a far better implementation,” and in the underwater domain, U2 reported receiving “feedback once a minute on all of the system health. So battery level, location, speed over ground... that's how we check and see now that it's doing what it's supposed to do.” As well as improving the operator's situational awareness, this would also reduce the mental demand required by the operator to diagnose and respond to an issue. For example, rather than simply reporting that a motor is overheating, the system could report that it is overheating because the operator's grip force is too strong, so that the operator would know how to resolve this. Wojtusch et al. (2019) also identified the importance of situational awareness, although did not specify a preference for how this should be addressed.

As was the case for the mandatory requirements, above, S1 did not highlight the system health monitoring as a high priority requirement. Again, here they were focused on functionality rather than system diagnostics, likely because of differences in their job role. If a surgeon's system stops working, a technician or the supplier will be responsible for fixing it. Although surgeons might not be responsible for diagnosing or resolving technical errors, S2 acknowledged that explainable system status monitoring functionality is still required for their system. S2 needed to be sure that “somebody who knows what they're doing with this thing can look at what it's actually doing vs. what it's meant to be doing... They need to tap into its entire operating system at the drop of the hat and the operating system needs to be configured in such a way that it's easily visible to a remote viewer or any viewer for that matter... because you have to be able to know what it thinks it's doing vs. what it's actually doing.” This assurance could be provided through the reputation of the system provider organization. In the other applications, however, the operator is also the engineer and technician, and, consequently, will be responsible for diagnosing and fixing faults. Having a more explainable system would, therefore, be more beneficial from their perspective.

##### 4.2.2.2. Facilitating operator training and familiarity

- Systems must be tested and trained upon with gradually increasing risk and realism, first in simulation, then on a physical mock up, and eventually *in-situ* in the real environment (R2).
- Operators must be given a large number of hours of practice and experience with a particular system that has a low frequency of faults and uncontrolled or unrequested movements (R5).

Trust is developed over time in both human-human teams (Rotter, 1971) and human-robot teams, and involves a consequence component (Hoff and Bashir, 2015). Training procedures for telemanipulation systems should, therefore, be designed to allow operators to build up this relationship. Increasing the risk and realism over the training process allows users to learn new skills and understanding of the system while maintaining an appropriate level of consequence. Importantly, training must focus not just on skills or techniques, but on operational time as well, as per the second requirement above. The two requirements are interlinked. By involving operators during the low risk testing stages, they can be provided with enough hours of meaningful practice to build their relationship with the system. The operator needs to have adequate experience witnessing the robot behaving as planned (i.e., building trust in the robot with no faults etc. that might damage trust). Furthermore, at a higher level, the organization would need to have adequate experience witnessing the operator achieving a low frequency of faults (building trust in the operator). These requirements could be satisfied for space systems, for example, by developing virtual and physical training environments on which the operators can train on Earth in a low risk setting will be essential for the developing an effective human-robot team.

#### 4.2.3. Medium-priority requirements

Of the remaining medium-priority requirements across-application (Table 7), R7 and R9 also fall into the “improving situational awareness” category, above. R6 and R8 can be categorized as “easing the operator's cognitive load.”

##### 4.2.3.1. Easing the operator's cognitive load

- Safety systems must trigger in a fail-safe mode when outside the system's capabilities (R6).
- Operators must have clear lines of communication with their support team to spread out responsibilities beyond the operator (R8).

The first of these requirements is a remote-system feature which ensures that, if safety thresholds are reached or the system fails, then it reverts to a safe state. This provides a reliable backup so that even if the system behaves unexpectedly, the operator has confidence that it will not have a negative impact.

The second requirement reduces the mental workload of the operator, which is a significant factor for users (Wojtusch et al., 2019). In addition to a human-robot team, these systems also involve several human-human teams which must be considered. Clear communication with the support team ensures that the operator receives the information they need at the right time. Additionally, if the support team are also trusted, the operator will have confidence that any tasks carried out in parallel will be completed as expected, without the need for supervision. Collectively, they can, therefore, operate more complex systems with greater confidence. As for the human-robot team, human-human trust with the support team must also be developed.

### 4.3. Practical implications of this study

The results of these interviews highlight the need for operator-centric approaches to demonstrate system trustworthiness, across telemanipulation applications. Examples of technical solutions suggested in the literature (Artigas et al., 2016; Panzirsch et al., 2020; Pryor et al., 2020) may be valuable in addressing the challenges posed by delayed telemanipulation, but in order to be used confidently, their capabilities must be gradually demonstrated to operators in scenarios with gradually increasing risk. Furthermore, new features should be explained to operators so that they understand their functionality, capabilities, and limitations. This could be achieved through first-hand practical experience, expert demonstration, or technical description.

### 4.4. Limitations of this study

Many of the requirements elicited here are operator-centric, which is a result of only interviewing operators. Although this fits within the scope of this study, the system should also be considered as a whole, capturing requirements from a range of perspectives. The requirements captured here may contradict those arising from a similar investigation with other stakeholders, for example, developers may propose technical features or high-level stakeholders may wish to apply policies to build trust. Future requirement elicitation studies would be needed to understand the perspectives of different stakeholders, before collating the results to understand the full picture. Furthermore, future work is needed to experimentally validate the impact of the requirements elicited in this study on system trustworthiness.

This study captured the requirements of the interviewees, given their experiences. However, user requirements are constantly evolving, and a future user may have a different background which produces different results. For example, they may be very experienced using augmented reality headsets, haptic gloves, or gesture control (Qi et al., 2021) technology, so may describe having these features as being similarly important to today's necessity for clear camera views. While the majority of requirements captured here are non-technical, we cannot discount the possibility that technical features may be necessary in improving trust for future users.

Another limitation of this study was the small number of interviews carried out for each domain. This problem is reduced, however, when collectively considering the responses of interviewees across domains to represent telemanipulation operators, in general. While increasing in prevalence, telemanipulation systems in industry are still uncommon across industries and hence, there is a small population of operators to draw from. It should also be noted that the value of this study lies in the collection of detailed qualitative data, across a series of 30–60 min long interviews (average 47 min) with subject matter experts, as opposed to short questionnaire responses. A further weakness of this study was the gender imbalance (1/13 female representation) within our sample. Although worldwide statistics of the gender ratio of teleoperators is difficult to find, the historical gender imbalance across engineering fields, surgery and the military likely impacted the number of female operators available for this study (Goldin, 1990; Jagsi et al., 2006; Sax et al., 2016). Using data from the BUPA website (BUPA, 2023), the gender ratio of RAS surgeons (consultants) in the UK is estimated to be 26 female to 351 male, i.e., 1/14 are female. Whilst it is certainly imbalanced, the ratio of female to male participants in our study seems representative of our target population.

## 5. Conclusions

This study aimed to identify how trust is built in teleoperated systems involving direct human-in-the-loop control of a robot manipulator. We explored this topic through interviews with operators from four well-established applications, with the intention of transferring techniques and features of existing systems to emerging applications in the space environment, in which operators will experience a noticeable and unavoidable delay. The results of these interviews led to the elicitation of a set of prioritized requirements which can be used across applications to develop trustworthy systems that involve direct telemanipulation.

Expert operators of telemanipulation systems, today, are capable of executing tasks under a range of challenging conditions, including delays comparable to Earth-Orbit and Earth-Moon communications. Even under these conditions, the operators trust their machines sufficiently to carry out the task. With the exception of surgery, the main reason for this is that they have a comprehensive understanding of their system's capabilities and limitations, from an engineering perspective. Developing this understanding must be a key user requirement for all direct telemanipulation systems, particularly in high-latency applications such as IOSM. High-priority requirements were also identified. As for in a team of humans, trust in a telemanipulation system must be developed over time based on positive experiences using the system. Confidence in the system's capabilities must be progressively inspired by demonstrating capabilities at increasing levels of risk, for example, beginning in simulation testbeds. Developing trust through technical features can be achieved by providing effective visual feedback systems which offer a range of viewpoints, in order to improve the operator's situational awareness.

## Data availability statement

Data are available at the University of Bristol data repository, data.bris, at https://doi.org/10.5523/bris.suauncyhoc1c2ahjpro04fwmy.

## Ethics statement

The studies involving humans were approved by Faculty of Engineering Research Ethics Committee of the University of Bristol. The studies were conducted in accordance with the local legislation and institutional requirements. The participants provided their written informed consent to participate in this study.

## Author contributions

JL, KE, JV, and AT contributed to conception of the study. JL and AT designed the study and acquired ethical approval. JL conducted the interviews, subsequent analysis, and wrote the first draft of the manuscript. AT and JV obtained the funding for this work. All authors contributed to manuscript revision, read, and approved the submitted version.
